# Predicting the Probability of the Incidence of Maxillary Sinus Fungus Ball in Patients Using Nomogram Models

**DOI:** 10.3390/diagnostics13193156

**Published:** 2023-10-09

**Authors:** Yu-Hsi Fan, Kai-Yi Shih, Pei-Wen Wu, Yen-Lin Huang, Ta-Jen Lee, Chi-Che Huang, Po-Hung Chang, Chien-Chia Huang

**Affiliations:** 1 Division of Rhinology, Department of Otolaryngology, Chang Gung Memorial Hospital, Chang Gung University, Taoyuan 333, Taiwana9665@cgmh.org.tw (P.-W.W.); entlee@cgmh.org.tw (T.-J.L.); hcc3110@cgmh.org.tw (C.-C.H.); bc1766@gmail.com (P.-H.C.); 2 School of Traditional Chinese Medicine, Chang Gung University, Taoyuan 333, Taiwan; kevin61007@gmail.com; 3 School of Medicine, Chang Gung University, Taoyuan 333, Taiwan; 4 Department of Anatomic Pathology, Chang Gung Memorial Hospital, Chang Gung University, Taoyuan 333, Taiwan; louisyhuang@gmail.com; 5 School of Medicine, National Tsing-Hua University, Hsinchu 300, Taiwan; 6 Institute of Stem Cell and Translational Cancer Research, Chang Gung Memorial Hospital, Taoyuan 333, Taiwan; 7 Department of Otolaryngology, Xiamen Chang Gung Hospital, Xiamen 361028, China

**Keywords:** computed tomography, fungus ball, maxillary sinus, nomogram, rhinosinusitis

## Abstract

Maxillary sinus fungal ball (MSFB) is the most common type of non-invasive fungal rhinosinusitis. Since MSFB requires a unique treatment strategy and is associated with potentially severe complications, timely and precise diagnosis is crucial. Computed tomography (CT) is the first-line imaging tool for evaluating chronic rhinosinusitis. Accordingly, we aimed to investigate the clinical and CT imaging characteristics of MSFB. We retrospectively enrolled 97 patients with unilateral MSFB and 158 with unilateral non-fungal maxillary rhinosinusitis. The clinical characteristics, laboratory data, and CT imaging features of participants were evaluated. Older age, female sex, lower white blood cell and neutrophil counts, and CT imaging features (including an irregular surface, erosion of the medial sinus wall, sclerosis of the lateral sinus wall, and intralesional hyperdensity) were significantly associated with MSFB. The presence of adjacent maxillary odontogenic pathology was associated with a decreased likelihood of the incidence of MSFB in unilateral maxillary rhinosinusitis. Separate nomograms were created for patients, without and with the use of CT scan, to predict the probabilities of MSFB in patients with unilateral maxillary rhinosinusitis. We proposed two nomograms based on the clinical and CT characteristics of patients with MSFB. These could serve as evaluation tools to assist clinicians in determining the need for undergoing CT and facilitate the accurate and timely diagnosis of MSFB.

## 1. Introduction

Fungal rhinosinusitis (FR) is defined as an infection arising from fungal sources that affect the paranasal sinuses and nasal cavities [1,2]. FR can be categorized into invasive and non-invasive FR. Invasive FR is highly uncommon and predominantly affects patients who are immunocompromised. Furthermore, it is often accompanied by severe complications and high mortality rates [3,4]. In contrast, most FR cases among individuals who are immunocompetent are non-invasive FR1 Fungal ball (FB), the most common subtype of non-invasive FR, consists of densely intertwined hyphae [5]. Notably, individuals with FB can develop invasive FR with severe orbital and intracranial complications, such as limited extraocular muscle movement, vision loss, and meningitis, with deteriorating host immunity [6].

The maxillary sinus is the site most commonly affected by FB. Maxillary sinus FB (MSFB) accounts for approximately 76.6–87.8% of cases of FB [5,7,8]. FB is characterized by the presence of yellowish/brownish clay-like or caseous matter [9], and FB affects only one side of the sinuses in most cases [5]. MSFB triggers mucosal inflammation and typically manifests with symptoms such as purulent nasal discharge, postnasal dripping, nasal obstruction, foul nasal smell, and facial pain, which are similar to those of non-fungal maxillary chronic rhinosinusitis [10].

Endoscopic sinus surgery (ESS) with the total removal of fungal material is the current treatment of choice for FB, as it typically has high eradication and low recurrence rates [5,7,8]. Nonetheless, owing to their remarkably similar clinical presentations, patients with MSFB could be misdiagnosed with non-fungal maxillary chronic rhinosinusitis and subjected to ineffective medical therapy. Considering the potentially severe complications and different treatment strategies required for MSFB, ensuring a timely and accurate diagnosis is crucial [11].

Several studies investigating the clinical characteristics of patients with MSFB have reported that older individuals and women are more predisposed to MSFB [8,11]. Computed tomography (CT) is the first-line imaging modality for evaluating paranasal sinus diseases because it provides high-resolution images of the anatomic structure surrounding the paranasal sinuses and the areas affected by the lesion [11]. Previous studies have explored the CT features of patients with MSFB [11,12]. Several CT imaging features, including surface irregularity, inner sinus wall erosion, lateral sinus wall sclerosis, and intralesional hyperdensity (IH), have been proposed to predict MSFBs [11]. Among these features, IH was reported to have the best specificity [12]. However, IH is not observed in approximately 20–30% of patients with MSFB. Consequently, we developed an imaging diagnostic algorithm for MSFB without IH and demonstrated its high sensitivity and specificity [11].

CT possesses good diagnostic value for MSFB; however, the decision to use CT for evaluating patients with suspected MSFB largely depends on the individual physician’s experience in clinical practice. Therefore, we aimed to investigate the clinical characteristics, laboratory data, and CT imaging features of patients with unilateral MSFB and developed two diagnostic models for MSFB: one for patients who had not yet undergone CT and another for those who had already undergone sinus CT. These models were created to assist clinicians in determining the necessity of performing additional CT scans and facilitate the accurate and timely diagnosis of patients with unilateral rhinosinusitis.

## 2. Materials and Methods

### 2.1. Study Participants

We identified 107 patients with histopathologically diagnosed MSFB between 1 January 2017 and 31 December 2018, by conducting an automated search within the histopathology database of our institute. After manually reviewing the preoperative paranasal sinus CT images, we excluded ten patients with bilateral maxillary sinus involvement. Thus, the remaining 97 patients diagnosed with unilateral MSFB were included in this study. Furthermore, 158 patients who underwent ESS to evaluate unilateral non-fungal maxillary rhinosinusitis (UMRS) during the same study period were recruited to the control group. All participants in the study underwent essential blood tests and CT of the paranasal sinuses without intravenous contrast enhancement as preoperative evaluations, and ESS with histopathological examination of the surgical specimens was performed. The study protocol was approved by the Institutional Review Board (approval number: 202001450B0). This study was performed in accordance with the relevant guidelines and regulations. The requirement for informed consent was waived owing to the retrospective nature of the study and the anonymization of the data. The STROBE reporting guidelines were adhered to.

### 2.2. Clinical Characteristics and CT Imaging Features

**Figure 1:**
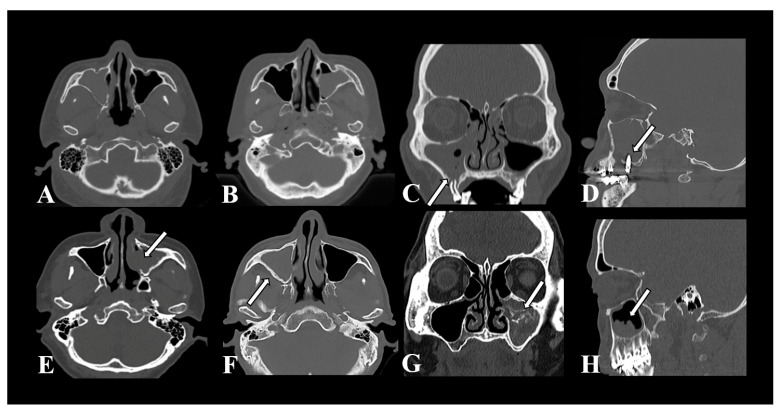
Computed tomographic imaging features of unilateral maxillary sinus lesions. (**A**) total opacification, (**B**) partial opacification, (**C**) periodontal bone loss, (**D**) penetrating dental implant, (**E**) erosion of the medial sinus wall, (**F**) sclerosis of the lateral sinus wall, (**G**) intralesional hyperdensity, and (**H**) irregular surface of the material.

Information regarding the age, sex, underlying conditions, clinical symptoms, laboratory data, and preoperative CT imaging features of the patients in the MSFB and UMRS groups was collected and analyzed. The CT imaging features adopted in this study were selected according to the findings of previous studies on FB imaging features [11]. Two physicians separately reviewed the preoperative CT images of the patients. They documented the imaging features, including total opacification, partial opacification, adjacent maxillary odontogenic pathology, erosion of the medial sinus wall, sclerosis of the lateral sinus wall, IH, and irregular surface of the material (Figure 1). Adjacent maxillary odontogenic pathology is defined as the presence of certain imaging features around the maxillary lesion, such as protrusion of the tooth root or dental implant, periodontal bone loss, periapical radiolucency, or the presence of an oroantral fistula, that supported its odontogenic origin. Erosion of the medial sinus wall is characterized by the absence of bone in the medial wall of the maxillary sinus [8,10]. Sclerosis of the lateral sinus wall refers to the ratio between the thickness of the lateral wall of the diseased sinus and that of its counterpart on the opposite side; this ratio exceeds 1.2. The thickness of the lateral wall is measured using axial CT images at the midpoint of the maxillary antral wall at the level where the inferior turbinate attaches to the maxillary sinus wall. IH is defined as the presence of calcifications or focal high-density lesions. Irregular surface of the material refers to a coarse or uneven surface observed in a maxillary sinus lesion.

### 2.3. Statistical Analysis

SPSS Statistics v26.0 (SPSS Inc., Chicago, IL, USA) and RStudio v2022.02.1 (RStudio, Boston, MA, USA) served as the statistical software in this study. Categorical variables were analyzed using the Chi-square test or Fisher’s exact test, whereas continuous variables were evaluated using Student’s *t*-test or the Mann–Whitney U test. Logistic regression analysis was performed to determine the associations between the various variables and MSFB. The odds ratios and 95% confidence intervals were calculated to assess the strength of the associations. Two nomograms were constructed to predict the probability of the incidence of MSFB. The predictive performance and reliability of the nomograms were further examined using receiver operating characteristic (ROC) curves, the area under the ROC curve (AUC), and calibration curves. Statistical significance was set at *p* < 0.05.

## 3. Results

### 3.1. Descriptive Characteristics of the Participants

Table 1 presents the demographic and clinical characteristics of the patients in the MSFB (*n* = 97) and UMRS (*n* = 158) groups. The mean age of the MSFB group was higher than that of the UMRS group (59.61 ± 13.2 years and 46.31 ± 14.8 years, respectively; *p* < 0.001). The number of female participants was 1.94-fold higher in the MSFB group, whereas the number of male participants was higher in the UMRS group (*p* < 0.001). A comparison between the preoperative laboratory data of the two groups revealed that white blood cell, neutrophil, and platelet counts were significantly lower in the MSFB group than those in the UMRS group (*p* < 0.001). No significant difference was observed between the two groups in terms of the proportion of patients with comorbid diabetes mellitus. Nasal obstruction was the only clinical symptom whose incidence differed significantly between the two groups. The prevalence of nasal obstruction was significantly higher in the UMRS group than that in the MSFB group (67.7% and 44.3%, respectively; *p* < 0.001). No statistically significant differences were observed in the proportion of patients with rhinorrhea, postnasal dripping, headache, facial pain, or hyposmia between the two groups.

### 3.2. Features of CT Imaging

Table 2 presents a comparison between the CT imaging features of the MSFB and UMRS groups. Irregular surface (*p* < 0.001), erosion of the medial sinus wall (*p* < 0.001), sclerosis of the lateral sinus wall (*p* < 0.001), and IH (*p* < 0.001) were significantly more prevalent in patients with MSFB than in those with UMRS. However, adjacent maxillary odontogenic pathologies were significantly more common in patients with UMRS.

### 3.3. Logistic Regression Analysis

The associations between the different variables and MSFB were evaluated using univariate regression analysis (Table 3). Older age (OR 1.07, 95% CI 1.04–1.09), female sex (OR 3.08, 95% CI 1.82–5.23), lower white blood cell count (OR 0.77, 95% CI 0.68–0.88), lower neutrophil count (OR 0.73, 95% CI 0.62–0.87), and lower platelet count (OR 0.55, 95% CI 0.36–0.86) were significantly associated with MSFB. Among the CT imaging features, MSFB exhibited significant associations with irregular surface of the material (OR 7.53, 95% CI 3.81–14.88), erosion of medial sinus wall (OR 14.66, 95% CI 3.81–14.88), sclerosis of lateral sinus wall (OR 4.40, 95% CI 2.05–9.44), and IH (OR 72.66, 95% CI 31.20–169.21). In contrast, the presence of a maxillary odontogenic pathology was associated with a decreased likelihood of the incidence of MSFB (OR 0.39, 95% CI 0.21–0.73).

### 3.4. Nomograms for Predicting the Probability of the Incidence of MSFB

**Figure 2:**
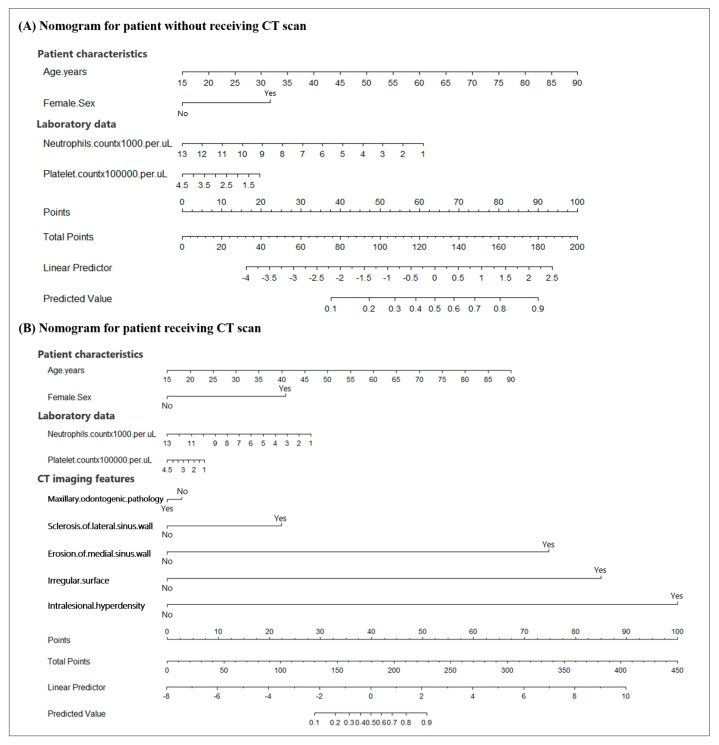
Nomograms for predicting the probability of the incidence of maxillary sinus fungal ball (MSFB) in patients without the use of CT scan (**A**) and patients with the use of CT scan (**B**). To use this nomogram, the corresponding position of each variable was identified first. Subsequently, a line was drawn vertically to the points axis to obtain the respective points. Lastly, the points from all seven variables were summed. A line was drawn from the corresponding position on the total points axis to the predicted value axis to determine the probability of the incidence of MSFB.

**Figure 3:**
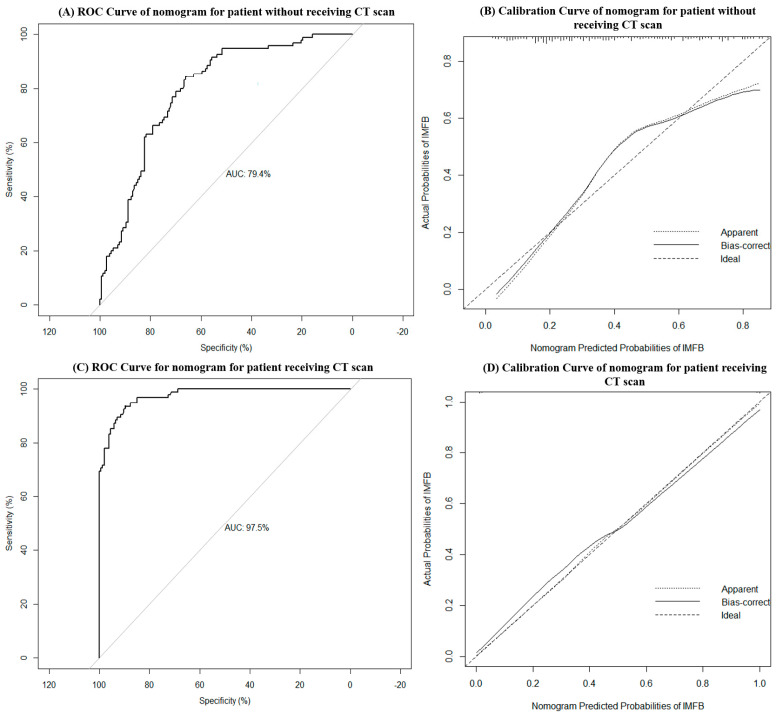
Receiver operating characteristic (ROC) curve of the nomogram model for predicting isolated maxillary sinus fungal ball (MSFB) in patients without the use of CT scan (**A**). The value of the area under the ROC curve (AUC) was 0.794 (95% confidence interval 0.738–0.850). On the calibration curve of the nomogram model for predicting MSFB in patients who had not undergone CT (**B**), the ideal line represents the ideal model that predicted the probabilities perfectly matching the actual probabilities. The apparent line and the bias-corrected line represent the nomogram model before and after incorporating the bootstrap resampling method, respectively. The ROC curve of the nomogram model in predicting isolated MSFB in patients with the use of CT scan (**C**). The value of AUC was 0.975 (95% confidence interval 0.960–0.990). On the calibration curve of the nomogram model for predicting MSFB in patients who had undergone CT (**D**), the ideal line represents the ideal model that predicted the probabilities perfectly matching the actual probabilities. The apparent line and the bias-corrected line, respectively, represent the nomogram model before and after incorporating the bootstrap resampling method.

Two nomograms were developed to predict the probability of the incidence of MSFB: one for patients without the use of CT scan and the other for patients with the use of CT scan (Figure 2A,B). To use these nomograms, clinicians had to first identify the appropriate position for each variable based on the clinical characteristics and CT imaging features of the patients. Subsequently, vertical lines were drawn towards the points axis to derive the corresponding points for each variable. The individual points for all the variables were summed to calculate the total points. Lastly, a vertical line was drawn downward from the corresponding position on the total points axis to the predicted value axis to determine the probability of the incidence of MSFB. The application of the nomograms is illustrated in the Supplementary Materials. AUC and calibration curves were used to validate the predictive performance and reliability of the nomograms. A higher AUC value indicated the superior predictive capability of the nomogram model. In the calibration curves, the ideal model was represented by a 45° diagonal line, indicating a perfect prediction of the observed outcomes. The two curves represent the unadjusted nomogram and bias-corrected models. The closer the matching of the three curves, the better the calibration of the nomogram model. The nomogram of the patients who had not undergone CT had an AUC of 0.794 (95% CI 0.738–0.850) (Figure 3A), with its calibration curve plotted in Figure 3B. By incorporating the CT imaging features as diagnostic variables, the AUC of the nomogram of patients who had undergone CT scans increased to 0.975 (95% CI 0.960–0.990) (Figure 3C), and its calibration curve also exhibited enhanced diagnostic accuracy (Figure 3D).

## 4. Discussion

MSFB is the most common non-invasive fungal sinusitis in clinical practice. It is characterized by the proliferation and aggregation of fungal hyphae in the maxillary sinus without local tissue invasion on histopathology [1]. Given the potential for the development of orbital complications of MSFB and the different treatment strategies required for its management, the timely and accurate diagnosis of MSFB is crucial [6,13]. Several studies have been conducted on MSFB, including studies on the diagnostic criteria based on the CT imaging features of MSFB [5,7,8,11,12]. However, the selection of patients for undergoing further CT examination remains a challenge. In the current study, we proposed the use of a nomogram as a preoperative diagnostic model of MSFB for patients without the use of CT scan (Figure 2A). A regression analysis revealed an AUC of 0.794 (Figure 3A). Clinicians can preliminarily estimate the probability of the incidence of MSFB in patients according to age, sex, and neutrophil and platelet counts, thereby facilitating a better assessment of the need for undergoing CT. Another nomogram was created for patients who had already undergone sinus CT. This nomogram incorporated the CT imaging features into the first nomogram (Figure 2B). The AUC increased to 0.975 (Figure 3C), indicating the high accuracy of the model in identifying patients with MSFB. Thus, these nomograms can facilitate the accurate and timely diagnosis of MSFB, thereby reducing unnecessary radiation exposure, medical costs, and potentially serious complications for patients.

Previous studies have linked older age and female sex with the incidence of MSFB [5,7]. In this study, the average age of patients with MSFB was 59.61 ± 13.2 years, which was significantly higher than that of the patients with UMRS (46.31 ± 14.8 years). A univariate regression analysis also demonstrated a significant association between older age and the incidence of MSFB, which was consistent with the findings of previous studies. This association may be attributed to age-related alterations in mucociliary clearance and immune system function, which can increase vulnerability to fungal infections [8]. Furthermore, older individuals tend to have increased medical visits, raising the likelihood of the diagnosis of MSFB. In the current study, the number of women was 1.94-fold higher among patients with MSFB. The factors contributing to the sex distribution disparity in FB may include the longer life expectancy of women and the potential involvement of female hormones in the pathogenesis of FR, as suggested by previous studies [14,15,16].

Diabetes mellitus (DM) impairs the immune system of patients and has been linked to the incidence of a wide range of infections, encompassing various fungal infections [3,8,17]. Previous studies identified DM as a common comorbidity in patients with FB.8 Similarly, we demonstrated the high prevalence of DM in both groups; DM was observed in 19.6% and 12.7% of the patients with MSFB and UMRS, respectively. However, no significant increase was observed in the risk of developing MSFB in patients with diabetes compared with the risk of developing UMRS. These results indicate that the sinuses of patients with diabetes are vulnerable to infection by fungi and other microbes. Nevertheless, further investigations are required to elucidate the relationship between DM and MSFB.

The current study is the first to report the use of white blood cells, neutrophils, and platelet counts in diagnosing MSFB. CRS is a heterogeneous disease divided into CRS without nasal polyps (CRSsNP) and CRS with nasal polyps (CRSwNP). Different types of white blood cells are involved in the development of these conditions. CRSsNP has long been considered a type 1 and type 3 inflammation and is mainly driven by neutrophils, whereas CRSwNP is thought to be strongly associated with type 2 inflammation [18,19]. As a prevalent condition of CRS, superimposed acute maxillary rhinosinusitis can also contribute to the activation and chemotaxis of neutrophils, consequently leading to an elevated peripheral blood neutrophil count. However, research focusing on the systemic immune profile of patients with FB remains scarce. Park et al. analyzed maxillary sinus lavage samples and mucosa from individuals with MSFB and reported that, despite the chronic fungal infection of the mucosa, only a mild inflammatory response was exhibited with no increase in the neutrophil count in the mucosa [20]. In addition, patients with sphenoid sinus FB were also found to have lower white blood cell counts than those with non-fungal sphenoid rhinosinusitis in a previous study [13]. We speculated that the distinct immune mechanisms involved in the two diseases may account for these disparities. The immune response triggered by MSFB may be less severe, leading to reduced levels of neutrophil production and chemotaxis, ultimately resulting in lower neutrophil and white blood cell counts in the peripheral blood. However, more comprehensive large-scale studies are necessary to validate our hypotheses.

Recent studies have highlighted that platelet functions are not only limited to hemostasis and thrombus formation but also play a pivotal role in the human immune response [21,22]. The immune system is activated during infection, and cytokines and inflammatory mediators that stimulate platelet production are released during inflammation. An elevated platelet count contributes to combating various infections (such as bacterial, viral, and fungal infections), facilitating pathogen clearance, and preventing pathogen spread within the body [22]. In the current study, patients with MSFB exhibited a significantly lower platelet count than those with UMRS. A lower platelet count was associated with MSFB in univariate regression analysis, which may be attributed to the differences in the immune mechanisms involved. Although evidence is limited, it is plausible that the immune response associated with MSFB may be less likely to induce platelet production and chemotaxis.

CT plays a vital role in the diagnosis and evaluation of sinus diseases. By offering high-resolution images and essential imaging information, CT enables physicians to establish accurate diagnoses and conduct comprehensive preoperative assessments of treatment plans. Previous studies have identified certain CT imaging characteristics, including material surface irregularity, medial sinus wall erosion, lateral sinus wall sclerosis, and IH, as predictors for MSFB [11,12]. The findings of the current study are consistent with these earlier observations. FB are formed by the aggregation and entwining of fungal hyphae, which leads to irregular or serrated projections on the lesion surface, as seen in CT images. As the fungus metabolizes, metal components accumulate within the hyphae, which present as high-density areas within the lesion on CT scans [23]. Local inflammatory responses induced by FB can trigger the remodeling of the bone surrounding the maxillary sinus, resulting in the bony dehiscence and/or sclerosis of the sinus wall [11,12].

A significant association was observed between the maxillary odontogenic pathology seen in CT images and UMRS in the univariate regression analysis in the current study. Odontogenic rhinosinusitis is a well-documented subtype of rhinosinusitis that stems from dental sources, representing approximately 10–40% of cases of chronic maxillary rhinosinusitis [24,25]. In the current study, 33.5% of the patients with UMRS exhibited adjacent odontogenic pathologies on CT images, which was higher than the proportion observed in the MSFB group (16.5%). Nonetheless, it is worth noting that the potential association between MSFB and dental pathologies and procedures has been widely discussed in numerous studies [8,10,26]. According to the literature, this association was primarily evaluated by the patients’ dental procedure history rather than their CT findings; however, the lack of a detailed history of dental procedures owing to the retrospective nature of the study design may have led to some limitations in the current study. However, we speculated that MSFB and UMRS have different degrees of association with adjacent maxillary odontogenic pathologies due to the high prevalence of odontogenic pathologies on CT images in the two groups.

This study has several limitations. All participants underwent ESS with histopathological diagnoses. Therefore, patients with mild symptoms or those ineligible for surgery may have been excluded, inevitably resulting in a certain degree of selection bias. Second, owing to the retrospective nature of the study design, patients with adjacent odontogenic pathologies were evaluated based on the CT findings without a detailed history of dental procedures. Thus, the association between dental problems and maxillary rhinosinusitis may have been underestimated. Lastly, this study focused on comparing MSFB and UMRS; however, many other lesions may exhibit comparable manifestations, such as mucoceles, neoplasms, and vascular lesions, which should be considered in daily clinical practice. Nonetheless, we believe that the proposed models would help clinicians differentiate the two most common inflammatory diseases in patients with unilateral maxillary sinus lesions.

## 5. Conclusions

We developed two nomograms based on the clinical and CT characteristics of patients with MSFB. These could serve as evaluation tools to assist clinicians in determining the need for undergoing CT and facilitate the accurate and timely diagnosis of MSFB.

## Figures and Tables

**Table 1:** Demographic and clinical characteristics of the study populations.

Variables	MSFB Group	UMRS Group	*p* Value
	(*n* = 97)	(*n* = 158)	
Age, years (mean ± SD)	59.61 ± 13.2	46.31 ± 14.8	<0.001 ***
Sex			
Male, *n* (%)	33 (34.0)	97 (61.4)	<0.001 ***
Female, *n* (%)	64 (66.0)	61 (38.6)	
Laboratory data			
WBC (×10^3^/μL)	6.69 ± 1.90	7.87 ± 2.41	<0.001 ***
Neutrophils count (×10^3^/μL)	3.97 ± 1.56	4.95 ± 2.13	<0.001 ***
Lymphocytes count (×10^3^/μL)	2.20 ± 0.722	2.27 ± 7.04	0.489
Eosinophils count (×10^2^/μL)	1.83 ± 1.91	1.78 ± 1.67	0.796
Platelet (×10^6^/μL)	2.50 ± 0.58	2.72 ± 0.63	0.006 **
Underlying condition			
Diabetes mellitus, *n* (%)	19 (19.6)	20 (12.7)	0.136
Clinical presentations			
Rhinorrhea, *n* (%)	60 (61.9)	113 (71.5)	0.109
Post nasal dripping, *n* (%)	47 (48.5)	85 (53.8)	0.407
Nasal obstruction, *n* (%)	43 (44.3)	107 (67.7)	<0.001 ***
Headache and facial pain, *n* (%)	28 (28.9)	43 (27.2)	0.775
Hyposmia, *n* (%)	25 (25.8)	42 (26.6)	0.887

MSFB, unilateral maxillary sinus fungal ball; UMRS, unilateral non-fungal maxillary rhinosinusitis; SD, standard deviation; WBC, white blood cell. ** *p* < 0.01, *** *p* < 0.001.

**Table 2:** Computed tomographic imaging features of MSFB and UMRS groups.

CT Imaging Features	MSFB Group	UMRS Group	*p* Value
	(*n* = 97)	(*n* = 158)	
Total opacification, *n* (%)	47 (48.5)	84 (53.2)	0.465
Partial opacification, *n* (%)	50 (51.5)	74 (46.8)	0.465
Irregular surface, *n* (%)	41 (42.3)	14 (8.9)	<0.001 ***
Maxillary odontogenic pathology, *n* (%)	16 (16.5)	53 (33.5)	0.003 **
Erosion of medial sinus wall, *n* (%)	53 (54.6)	12 (7.6)	<0.001 ***
Sclerosis of lateral sinus wall, *n* (%)	88 (90.7)	109 (69.0)	<0.001 ***
Intralesional hyperdensity, *n* (%)	79 (81.4)	9 (5.7)	<0.001 ***

CT, computed tomographic; MSFB, unilateral maxillary sinus fungal ball; UMRS, unilateral non-fungal maxillary rhinosinusitis. ** *p* < 0.01, *** *p* < 0.001.

**Table 3:** Logistic regression analyses of the associated variables of MSFB.

Variables	Univariate Regression Analysis	
	Odds Ratio (95% CI)	*p* Value
Characteristics of patients		
Age (years)	1.07 (1.04−1.09)	<0.001 ***
Diabetes mellitus	1.68 (0.85−3.33)	0.138
Female sex (female versus male)	3.08 (1.82−5.23)	<0.001 **
Laboratory data		
WBC count (×10^3^/μL)	0.77 (0.68−0.88)	0.001 **
Neutrophils count (×10^3^/μL)	0.73 (0.62−0.87)	<0.001 ***
Lymphocytes count (×10^3^/μL)	0.88 (0.61−1.27)	0.480
Eosinophils count (×10^2^/μL)	1.00 (0.99−1.02)	0.761
Platelet (×10^6^/μL)	0.55 (0.36−0.86)	0.008 **
CT imaging features		
Total opacification	0.83 (0.50−1.37)	0.465
Partial opacification	1.21 (0.73−2.00)	0.465
Maxillary odontogenic pathology	0.39 (0.21−0.73)	0.003 **
Irregular surface	7.53 (3.81−14.88)	<0.001 ***
Erosion of medial sinus wall	14.66 (7.19−29.85)	<0.001 ***
Sclerosis of lateral sinus wall	4.40 (2.05−9.44)	<0.001 ***
Intralesional hyperdensity	72.66 (31.20−169.21)	<0.001 ***

MSFB, unilateral maxillary sinus fungal ball; CI, confidence interval; WBC, white blood cell; CT, computed tomography. ** *p* < 0.01, *** *p* < 0.001.

## Data Availability

Not applicable.

## Supplementary Materials

The following supporting information can be downloaded at: https://www.mdpi.com/article/10.3390/diagnostics13193156/s1, Figure S1: Steps for using a nomogram to predict the probability of a maxillary sinus fungal ball (MSFB) for a patient without receiving CT scan; Figure S2. Computed tomographic features of the patient’s maxillary sinus lesion. (A) Partial opacification, (B) irregular surface, and (C) sclerosis of the lateral sinus; Figure S3. Employing the nomogram to the patient after receiving CT scan. By this nomogram, the patient’s probability of having a MSFB was estimated to be around 80%.
