# Supplementary material for: Predicting the Probability of the Incidence of Maxillary Sinus Fungus Ball in Patients Using Nomogram Models
Source: Diagnostics (Basel). 2023 Oct 9;13(19):3156. doi: 10.3390/diagnostics13193156 (PMC10572425; doi:10.3390/diagnostics13193156)
Supplement: Supplementary file 1 [file diagnostics-13-03156-s001.zip › diagnostics-2583650-supplementary.pdf]

diagnostics

MDPI

Supplementary

Predicting the Probability of the Incidence of Maxillary Sinus Fungus Ball in Patients Using Nomogram Models

*Employing nomograms to predict the probability of a patient with maxillary sinus fungus ball (MSFB)

Prior to employing the nomogram models, it's essential to collect key patient data, such as age, gender, neutrophil count, and platelet count. Following this, depending on whether the patient has undergone a sinus computed tomography (CT) scan, the suitable nomogram should be chosen to determine the patient's probability of having a MSFB. For the purpose of illustrating the use of our presented nomograms, a participant from the study was selected as a representative case. A 67-year-old female visited our outpatient clinic, presenting symptoms of rhinorrhea and post nasal dripping persisting for 5 months. Her neutrophil count was recorded as 3,077 per μL and her platelet count was recorded as 331,000 per μL. Utilizing this data, we used the first nomogram to preliminarily estimate the probability of MSFB for this patient, following the steps in **Supplementary Figure S1**, which was **approximately 72%.** The patient subsequently underwent a sinus CT scan without contrast, uncovering a partially opacified lesion in her left maxillary sinus. Although no intralesional hyperdensity was observed, irregular surface and sclerosis of the lateral sinus wall were seen on her CT images (**Supplementary Figure S2**). With the CT images, we applied the second nomogram to further determine the patient's probability of having MSFB, which was **estimated to be around 80%** (**Supplementary Figure S3**).

<u>Step 1:</u> Find the corresponding position of each variable according to the patient's clinical information.

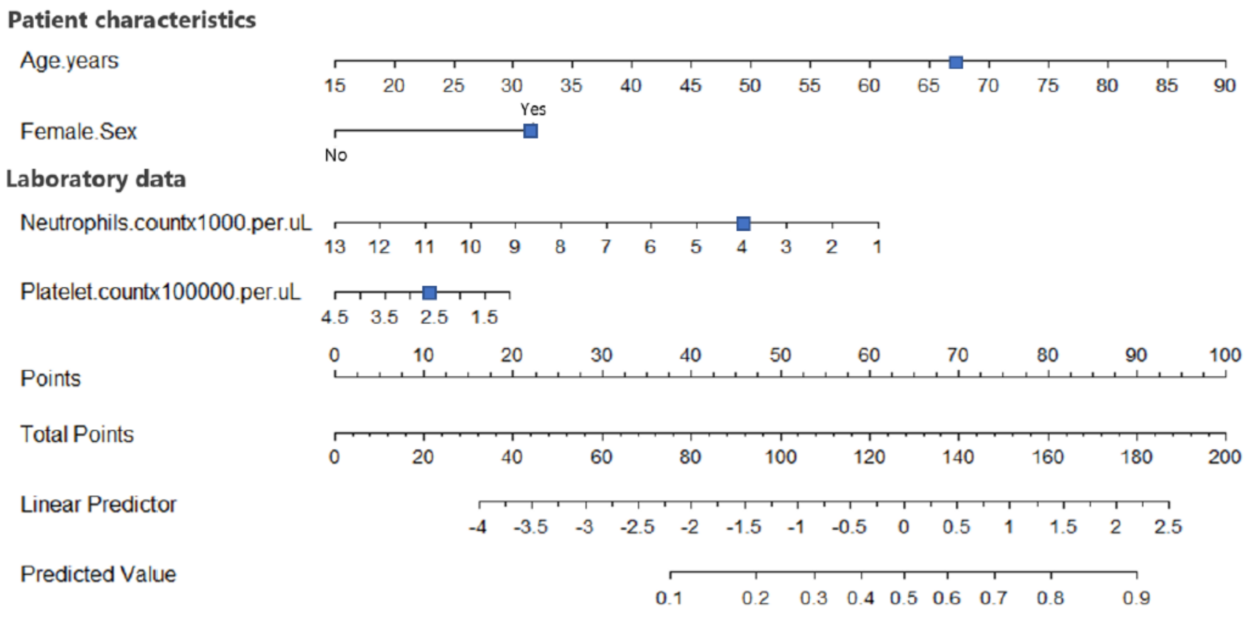

<u>Step 2:</u> Draw a vertical line downward to the Points axis to obtain the respective points for each variable. Subsequently, compute the total points by summing up the respective points for each variable. For this patient, the total points amount to ***151 points***.

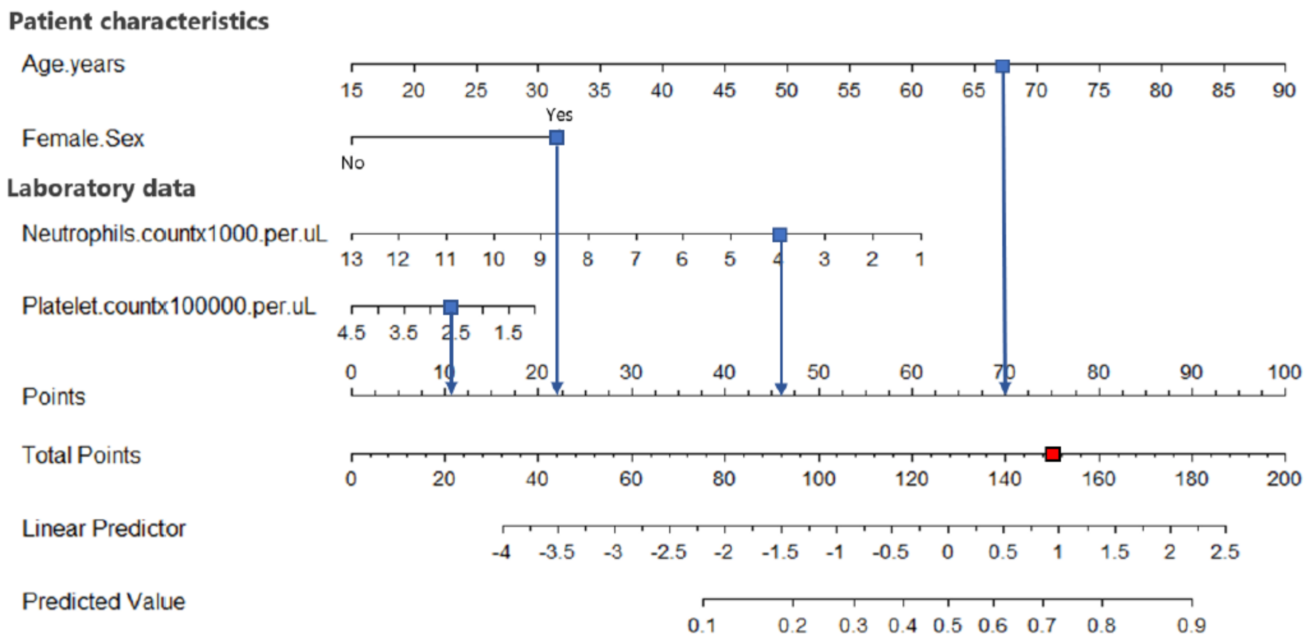

<u>Step 3:</u> Lastly, from the corresponding position on the Total Points axis, draw a vertical line downward to the Predicted Value axis to determine the probability of SSFB. The probability of MSFB for this patient ***<u>was about 72%</u>*** by the nomogram.

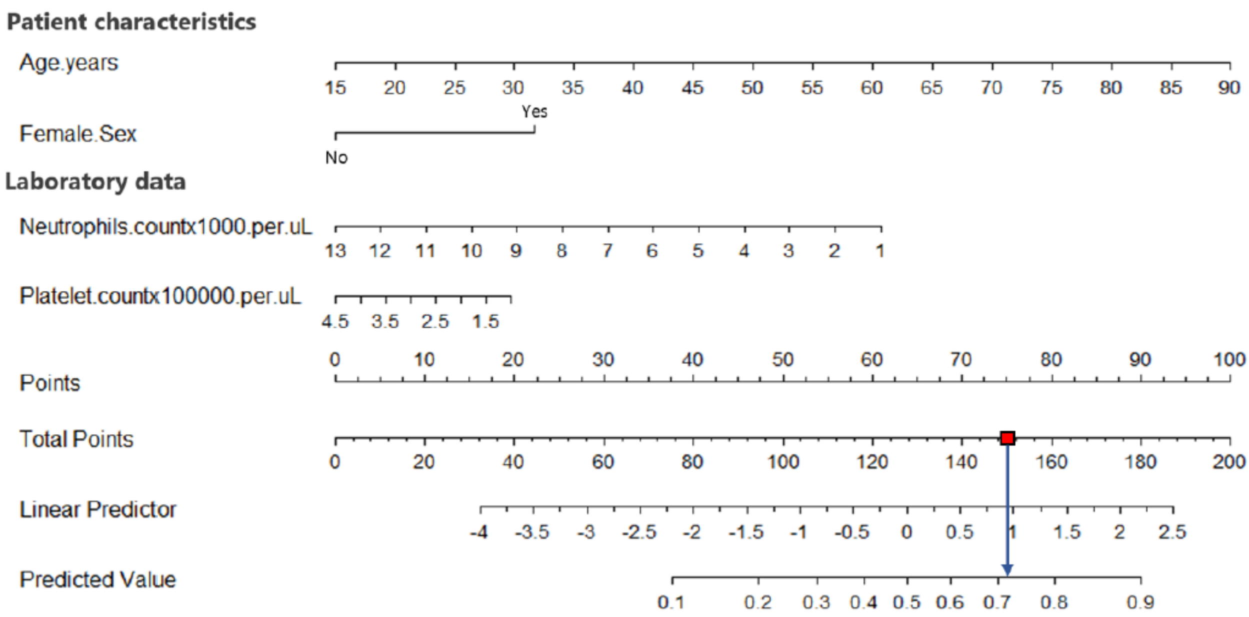

Supplementary Figure S1. Steps for using a nomogram to predict the probability of a maxillary sinus fungal ball (MSFB) for a patient without receiving CT scan.

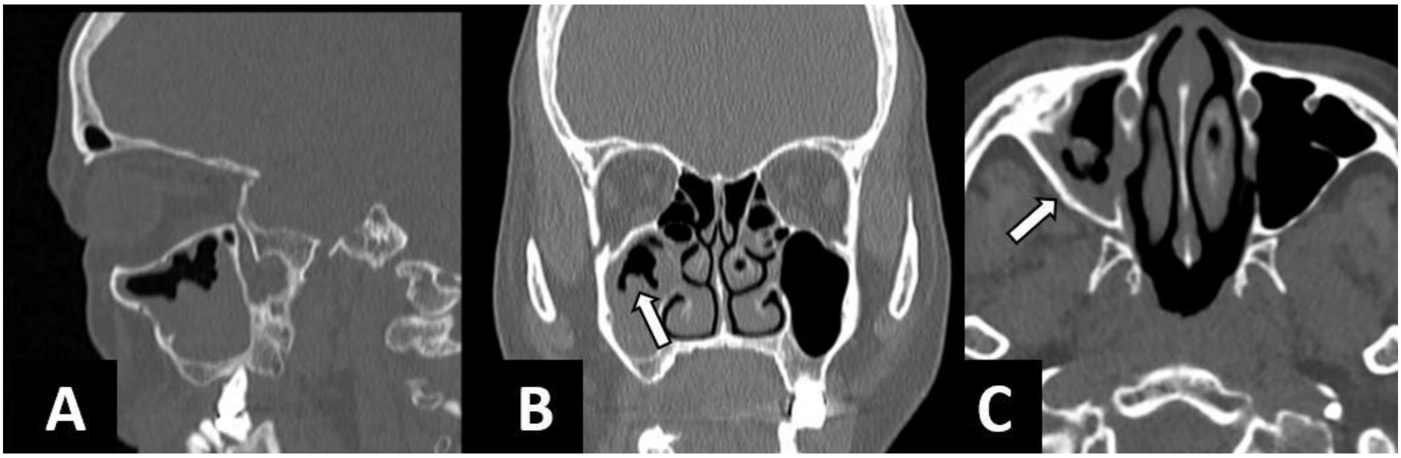

Supplementary Figure S2. Computed tomographic features of the patient's maxillary sinus lesion. (**A**) Partial opacification, (**B**) irregular surface, and (**C**) sclerosis of the lateral sinus.

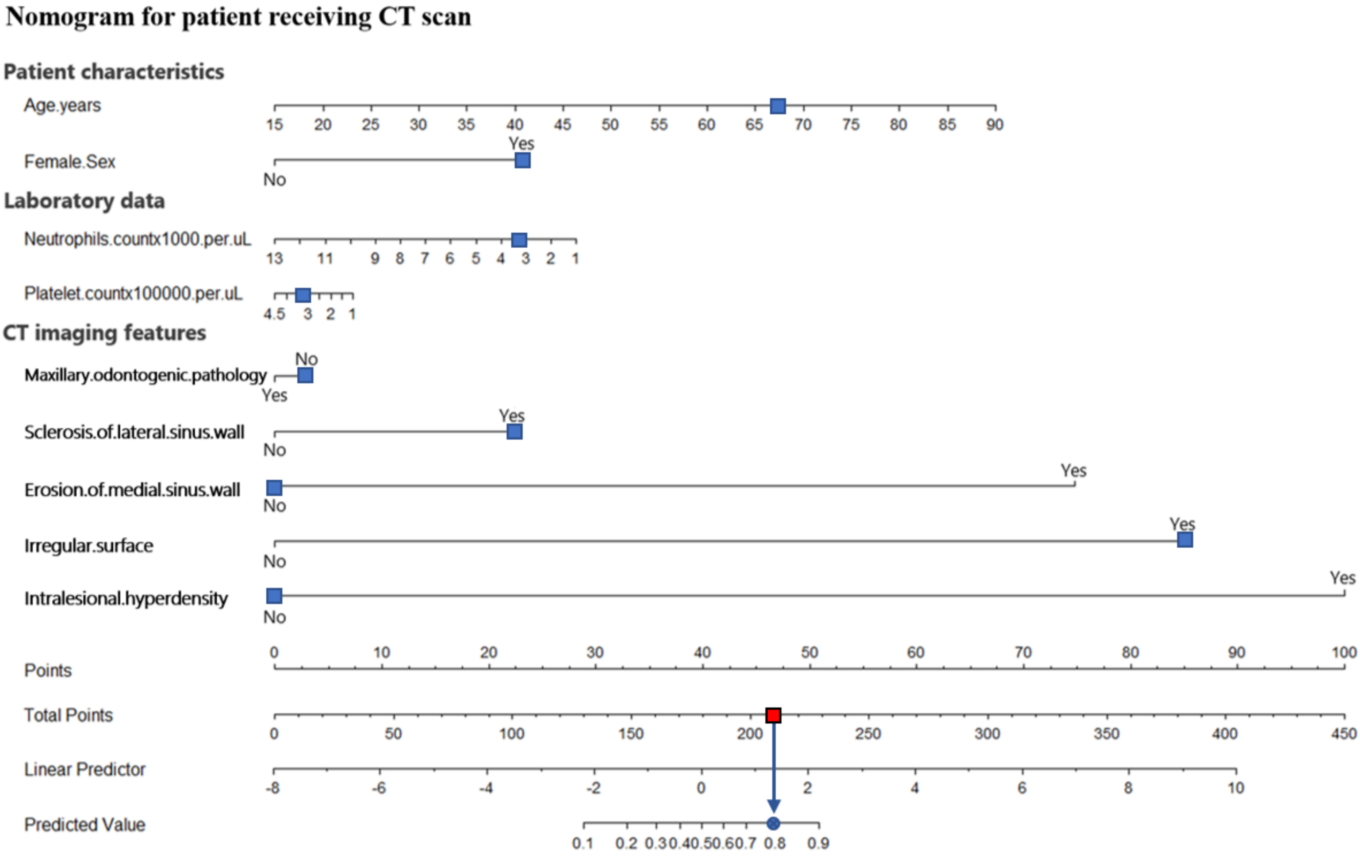

Supplementary Figure S3. Employing the nomogram to the patient after receiving CT scan. By this nomogram, the patient's probability of having a MSFB was estimated to be **around 80%.**
